# Harnessing Cytosine for Tunable Nanoparticle Self-Assembly Behavior Using Orthogonal Stimuli

**DOI:** 10.1021/acs.biomac.4c00352

**Published:** 2024-07-15

**Authors:** Sam J. Parkinson, Stephen D. P. Fielden, Marjolaine Thomas, Alisha J. Miller, Paul D. Topham, Matthew J. Derry, Rachel K. O’Reilly

**Affiliations:** † School of Chemistry, University of Birmingham, Birmingham, Edgbaston B15 2TT, United Kingdom; ‡ Aston Institute for Membrane Excellence, Aston University, Birmingham B4 7ET, United Kingdom

## Abstract

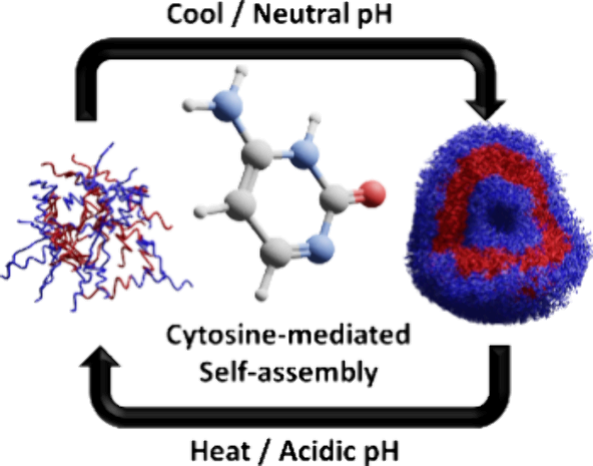

Nucleobases control the assembly of DNA, RNA, etc. due to hydrogen bond complementarity. By combining these unique molecules with state-of-the-art synthetic polymers, it is possible to form nanoparticles whose self-assembly behavior could be altered under orthogonal stimuli (pH and temperature). Herein, we report the synthesis of cytosine-containing nanoparticles via aqueous reversible addition-fragmentation chain transfer polymerization-induced self-assembly. A poly(*N*-acryloylmorpholine) macromolecular chain transfer agent (mCTA) was chain-extended with cytosine acrylamide, and a morphological phase diagram was constructed. By exploiting the ability of cytosine to form dimers via hydrogen bonding, the self-assembly behavior of cytosine-containing polymers was altered when performed under acidic conditions. Under these conditions, stable nanoparticles could be formed at longer polymer chain lengths. Furthermore, the resulting nanoparticles displayed different morphologies compared to those at pH 7. Additionally, particle stability post-assembly could be controlled by varying pH and temperature. Finally, small-angle X-ray scattering was performed to probe their dynamic behavior under thermal cycling.

## Introduction

Nature displays much greater control over self-assembly on the molecular and macromolecular level than the synthetic chemist can currently achieve. Self-assembly of nucleic acids (DNA and RNA) exploits the specific hydrogen bonding interactions between nucleobase pairs (adenine/thymine and cytosine/guanine) for biological encoding and stereospecific molecular assembly. Inspired by these natural systems, chemists have combined synthetic polymers with nucleobases for various purposes, such as to control polymer tacticity,^1^ dictate polymer sequence,^2^ template polymerizations,^3−5^ and enhance adhesive properties.^6,7^

Nucleobases have also been used to control the self-assembly of polymeric nanoparticles.^8−12^ Typically, this has been achieved either by modifying polymer end-groups with complementary nucleobases or polymerizing monomers containing nucleobase functionalities. The assembly of these polymers has predominantly been performed using postpolymerization techniques such as solvent switching or nanoprecipitation, and various morphologies such as spheres,^5^ worms,^13^ vesicles,^14^ and spindles^15^ have been observed. An alternative to post-polymerization self-assembly techniques is to perform polymerization heterogeneously. One of the most powerful techniques in heterogeneous polymerization is polymerization-induced self-assembly (PISA).^16^ By performing the polymerization of nucleobase monomers under PISA, it can be expected to influence the self-assembly process. We reported the RAFT dispersion polymerization of thymine- or adenine-containing monomers using a poly(methyl methacrylate) macromolecular chain transfer agent (mCTA) in either chloroform or 1,4-dioxane.^17,18^ Altering the solvent and polymer chain length and therefore the solubility of the resultant polymer block significantly affected the nanoparticle morphology obtained, with spheres, worms, vesicles, or disk-like nanoparticles being observed.

Nucleobase interactions have also been shown to affect nanoparticle morphology via postassembly modifications.^10,12,19−21^ We have previously reported a morphological transformation process where a spherical micelle containing thymine within the core was elongated into anisotropic worms of a controlled length via the addition of a specified amount of linear polymers containing the complementary nucleobase.^12^ This approach can also be used to control the growth of nodes from polymersomes.^22^ Yan et al. have also demonstrated a similar transformation, a postself-assembly morphological transformation driven by nucleobase interactions.^19^

While most of the research exploiting nucleobase interactions in polymers primarily focuses on the adenine-thymine pair, there is limited research using cytosine or guanine, even though it exhibits stronger binding due to the increased number of hydrogen bonding sites.^15,23,24^ This is likely due to the difficulty associated with the solubility of guanine.^25^ Additionally, guanine structures are also known to self-assemble and form various architectures such as quartets,^26^ G-quadruplexes,^27^ and ribbon-like structures,^28^ which may also complicate their incorporation into polymers. However, cytosine has a higher water solubility than guanine, and at low pH, the partial protonation of cytosine causes it to dimerize forming an i-motif. This can produce four-stranded DNA assemblies, analogous to G-quadruplexes.^29^ Therefore, it can be expected that incorporating cytosine into polymers would be a convenient way to endow assemblies with pH-responsive behavior. These structures are well known in nucleic acid research and are often used to control secondary structures. It is thus likely that incorporating these nucleobases into polymers will modify their self-assembly behavior.

Herein, we report the synthesis of cytosine-containing nanoparticles via aqueous reversible addition-fragmentation chain transfer polymerization-induced self-assembly (RAFT-PISA). Poly(*N*-acryloylmorpholine) mCTA was chain-extended with a cytosine-based acrylamide monomer, and a morphological phase diagram was constructed. The self-assembly behavior of cytosine-containing polymers under acidic conditions, where i-motif formation is possible, was monitored, and the resulting nanoparticles displayed thermo-responsive behavior. The stimuli-responsive self-assembly of these nanoparticles during temperature cycling was then probed using *in situ* small-angle X-ray scattering (SAXS) to assess its dynamic behavior.

## Experimental Section

### Materials

2,2′-Azo-bis(isobutyronitrile) (AIBN) was obtained from Molekula and recrystallized from methanol. 2,2′-Azo-bis[2-(2-imidazolin-2-yl)propane]dihydrochloride (VA-044, Wako) was used without further purification. Cytosine was purchased from Acros. Sodium hydride (60% dispersion in mineral oil) and 4-acryloylmorpholine (NAM) were purchased from Sigma-Aldrich. Triethylamine was received from Fisher Chemical and used without any purification. Acryloyl chloride and pyridine were used as received from Sigma-Aldrich. Dialysis membranes (MWCO = 3.5 kDa) were purchased from Spectra/Por. DMF, DMSO, and other chemicals were obtained from Fisher Chemicals and used without further purification. Dry solvents were obtained by passing over a column of activated alumina using an Innovative Technologies solvent purification system. 2-(((Butylthio)carbonothiolyl)thio)propanoic acid was synthesized according to the literature procedure previously used in our group.^12^

### ^1^H NMR Spectroscopy

^1^H NMR spectra were recorded at 300 MHz on a Bruker DPX-400 spectrometer in either D_2_O for macro-CTA synthesis or (CD_3_)_2_SO for all small molecule synthesis and polymerizations.

### Size Exclusion Chromatography (SEC)

SEC measurements were performed on an Agilent 1260 Infinity detector suite system fitted with refractive index (RI) and ultraviolet (UV) detectors (λ = 309nm) equipped with a PLPolarGel 3 μm (50 × 7.5 mm) guard column and two PLPolarGel 5 μm (300 × 7.5 mm) columns using DMSO with 0.1% w/v LiBr at 50 °C as the eluent at a flow rate of 1.0 mL min^–1^. SEC data was calibrated against polymethylmethacrylate standards.

### Dynamic Light Scattering (DLS)

Hydrodynamic diameters (*D*_h_) and size distributions of the self-assemblies were determined by a Malvern Zetasizer NanoZS instrument operating at 25 °C with a 4 mW He-Ne 633 nm laser module. Measurements were made at a detection angle of 173° (back scattering), three runs for each sample, and Malvern DTS 6.20 software was used to analyze the data.

### Transmission Electron Microscopy (TEM)

TEM was performed using a JEOL 1400 instrument at 200 kV. TEM solutions were typically made up at 0.1 mg mL^–1^ in water. Then, 10 μL of sample solution was dropped onto a carbon/Formvar-coated copper grid placed on filter paper. After removing excess liquid, 10 μL of a 1% uranyl acetate solution was dropped onto the grid, and excess liquid was removed. The grids were left to dry before being loaded onto the microscope.

### Small-Angle X-ray Scattering (SAXS)

SAXS patterns were recorded at a synchrotron source (Diamond Light Source, station I22, Didcot, UK^36^; experiment ID SM33098) using monochromatic X-ray radiation (X-ray wavelength λ = 1.00 Å, with scattering vector *q* ranging from 0.0017 to 0.17 Å^–1^, where *q* = 4π sin θ/λ and θ is one-half of the scattering angle) and a 2D Pilatus 2 M pixel detector (Dectris, Switzerland). All static SAXS measurements were performed on 2.5% w/w copolymer dispersions in 2.0 mm diameter polycarbonate capillaries. Scattering data were reduced and normalized, with glassy carbon being used for the absolute intensity calibration utilizing standard routines available at the beamline^50,51^ and further analyzed (background subtraction and data modeling) using Irena SAS macros for Igor Pro.^52^

### Synthesis of Cytosine Acrylamide (CAm)

To a suspension of cytosine (5.0 g, 45 mmol) in dry DMF (100 mL) was slowly added 60% NaH dispersed in mineral oil (2.0 g, 50 mmol NaH) in small portions under a nitrogen atmosphere. The mixture was stirred for 1 h, until no gas was produced. The viscous mixture was immersed into an ice bath and *N*-(3-bromopropyl)acrylamide freshly synthesized (8.6 g, 45 mmol) was added dropwise. The ice bath was left in place, and the yellow viscous mixture was stirred overnight. The resulting suspension was concentrated under a high vacuum at 50 °C to give a highly viscous oil. The crude residue was further purified by column chromatography using a mixture of CH_2_Cl_2_ and CH_3_OH as an eluent and gradient from 1:0 to 8:2 to give a white solid, CAm (3.18 g, 52%). ^1^H NMR (400 MHz, DMSO-*d*_6_) δ 8.12 (t, *J* = 5.7 Hz, 1H), 7.58 (d, *J* = 7.1 Hz, 1H), 7.09–6.83 (m, 2H), 6.20 (dd, *J* = 17.1, 10.0 Hz, 1H), 6.07 (dd, *J* = 17.1, 2.3 Hz, 1H), 5.63 (d, *J* = 7.1 Hz, 1H), 5.58 (dd, *J* = 10.0, 2.4 Hz, 1H), 3.64 (t, *J* = 6.9 Hz, 2H), 3.11 (m, 2H), 1.73 (tt, *J* = 7.0 Hz, 2H)

### Synthesis of Poly(*N*-acryloyl morpholine)_40_ (PNAM_40_) mCTA

A typical synthesis of a PNAM_40_ macro-CTA was as follows: *N*-acryloyl morpholine (10 g, 70 mmol, 40 equiv), PATBC (0.42 g, 1.7 mmol 1 equiv), and AIBN (0.03 g, 170 μmol 0.1 equiv) were added to a round-bottom flask and dissolved in 1,4-dioxane (24 mL) to give a 30% w/w reaction solution. A stirrer bar was added, and then, the flask was sealed and sparged with nitrogen for 20 min. The sealed flask was then immersed in an oil bath at 70 °C and left for 5 h after which it was removed from the oil bath and quenched by exposure to oxygen. Samples were then taken for ^1^H NMR and SEC (*M*_n_ = 7.8 kDa, *Đ*_M_ = 1.12) analysis followed by purification by precipitation in diethyl ether to yield a yellow powder.

### Synthesis of Diblock Copolymer Nanoparticles

A typical synthetic procedure to achieve PNAM_40_-*b*-PCAm_200_ nanoparticles at 10% w/w solids was as follows: PNAM_40_ macro-CTA (9 mg, 1.59 μmol, 1 equiv), CAm (80 mg, 317 μmol, 200 equiv), and VA-044 (0.10 mg, 0.32 μmol, 0.2 equiv) (a stock solution of 2 mg of VA-044 in 1 mL of water was made prior; a volume of 51.5 μL was taken and added to the reaction mixture) were dispersed in deionized water (0.89 mL) and sealed in a 7 mL vial bearing a magnetic stirrer bar. The resulting monomer-in-water solution was degassed by sparging with N_2_ for 15 min. The sealed vial was heated at 50 °C with magnetic stirring for 2 h to ensure full monomer conversion. After this period, the reaction mixture was exposed to air and cooled to room temperature. ^1^H NMR in DMSO-*d*_6_ and SEC analyses in DMSO + 0.1% w/w LiBr of the pure diblock copolymers were performed after the reaction. DLS analysis and dry-state TEM imaging were performed on samples after dilution to an appropriate analysis concentration.

## Results and Discussion

**Figure 1:**
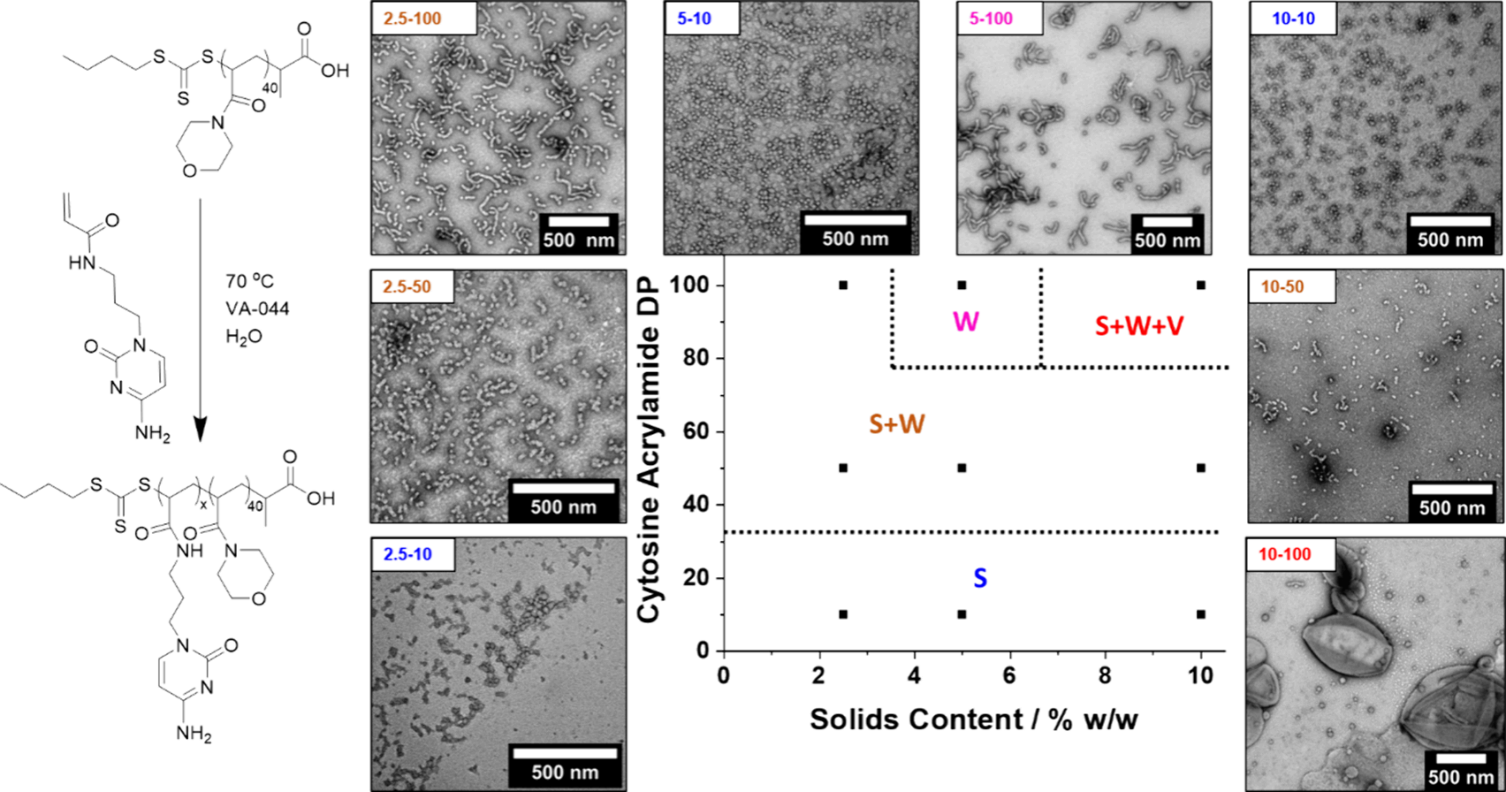
Reaction scheme (left) and representative transmission electron microscopy images (right) showing sphere, worm, and vesicle morphologies obtained from PNAM_40_*-b-*PCAm_*x*_ diblock copolymer nano-objects at pH 7. S = spheres (blue), S + W = mixed spheres and worms (orange), W = worms (pink), S + W + V = mixed spheres, worms, and vesicles (red). All reactions were conducted at 50 °C with total solid concentrations of 2.5, 5, or 10% w/w and [CAm]:[PNAM mCTA]:[VA-044] = 10/50/100:1:0.2. Notation in the corner of TEM images indicates % w/w and DP.

A poly(acryloyl morpholine) (PNAM) mCTA (*M*_n_ = 7.8 kDa, *Đ*_M_ = 1.12) was synthesized via RAFT solution polymerization with a mean degree of polymerization (DP) of 40, as determined by ^1^H NMR, and was then subsequently chain-extended via RAFT aqueous dispersion polymerization with cytosine acrylamide (CAm) in pure water at pH 7. A full phase diagram was constructed at 50 °C, total solid concentration was 2.5, 5, or 10% w/w, and [CAm]:[PNAM mCTA]:[VA-044] = 10/50/100:1:0.2, to understand self-assembly behavior in water—a solvent that is competitive to nucleobase hydrogen bonding (Figure 1 and Figure S1 and Table S1). For PNAM_40_*-b-*PCAm_*x*_ with a low PCAm DP (*x* = 10), spheres were observed. As the PCAm DP was increased (*x* = 50), a mixed sphere and worm phase were observed. To obtain a pure worm phase, the PCAm DP (*x* = 100) and solid content needed to be increased (5% w/w). A further increase in the solid content (10% w/w) led to the formation of a mixed phase containing spheres, worms, and vesicles. While similar mixed phases have been reported in the literature,^30^ it seems rather counterintuitive for these three morphologies to coexist at a given polymer composition due to the different molecular curvatures required for each morphology. However, the relatively high polymer dispersity (*Đ*_M_= 1.35) confers a significant compositional heterogeneity.^31^ It has been shown that different-sized polymer chains can undergo narcissistic self-sorting during nanoparticle formulation.^32^ Thus, more PNAM-rich copolymer chains should tend to form spheres, while the fraction of chains that are PCAm-rich should favor vesicles, and intermediate copolymer compositions should produce worms. Finally, it is worth noting that under these polymerization conditions (pH = 7), when targeting a PCAm DP greater than 100, precipitation was observed at all solid contents. This behavior was likely due to the formation and subsequent aggregation of colloidally unstable nanoparticles.

**Figure 2:**
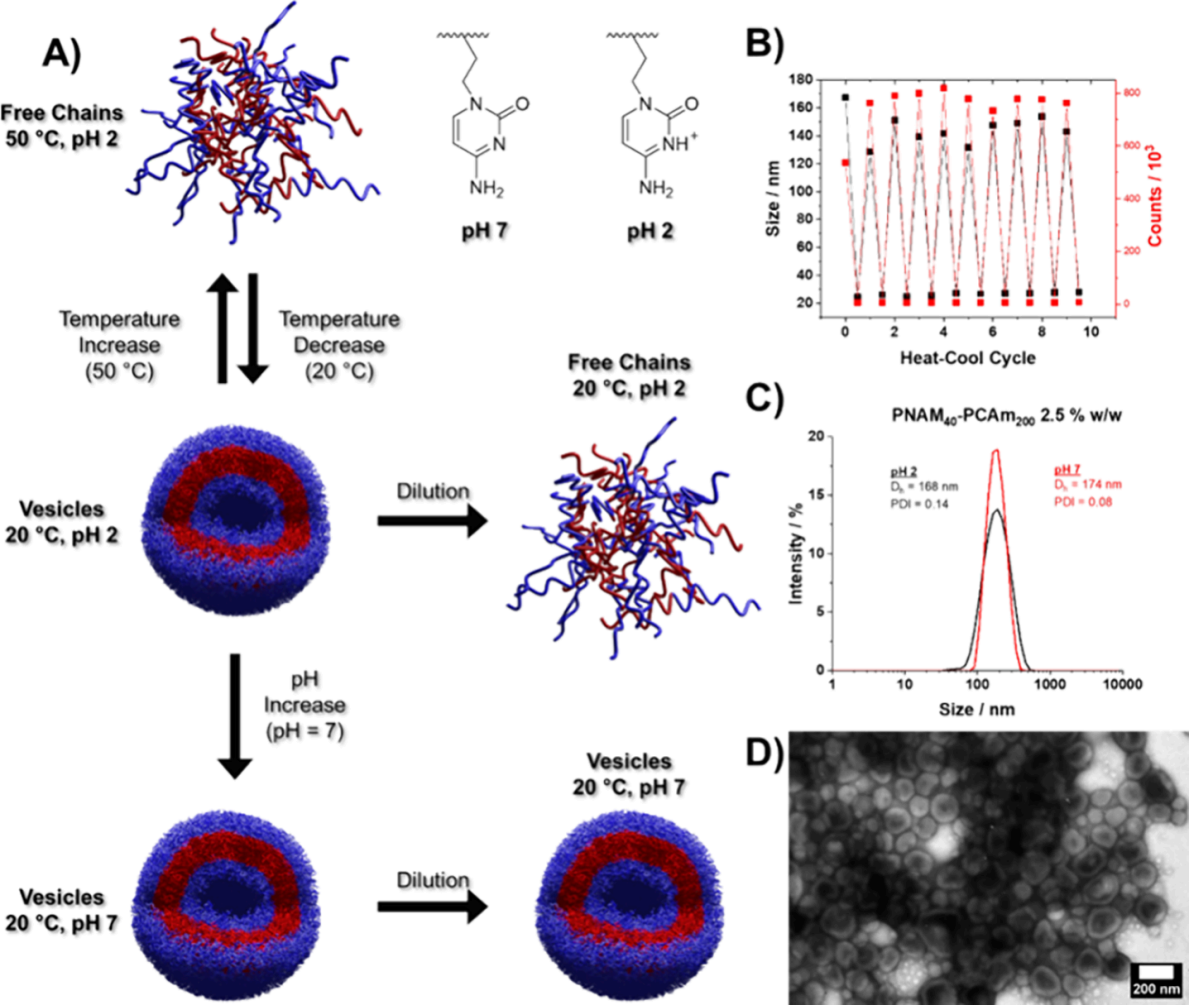
(A) Schematic illustrating the thermo-responsive self-assembly of cytosine-containing diblock copolymers under acidic conditions followed by morphological trapping upon basification. (B) DLS size analysis of PNAM_40_*-b-*PCAm_200_ nanoparticles at pH 2 during thermal cycling between 50 and 20 °C. (C) DLS traces for PNAM_40_-*b*-PCAm_200_ nanoparticles before (pH 2) and after basification (pH 7). (D) Representative transmission electron microscopy image of PNAM_40_*-b-*PCAm_200_ nanoparticles after basification to pH 7.

Given that cytosine assembly is known to be pH-responsive,^29^ we wanted to explore whether this would translate to polymer assembly. As the PCAm chains need to be protonated to form i-motifs, next, we performed the chain extension of PNAM mCTA with CAm under acidic conditions. The solution pH (pH = 2) was significantly below the p*K*_a_ of the N-3 amine (p*K*_a_ = 4.2).^33^ Initially, a range of different PCAm DPs was targeted (DP = 50, 100, 150, and 200) with a [PNAm mCTA]:[VA-044] = 1:0.2 and solid concentration of 2.5% w/w to determine whether it was possible to exceed the chain length limit we had previously seen under neutral conditions. ^1^H NMR spectroscopy indicated that all polymerizations reached full conversion. However, all polymer solutions were completely transparent upon removal from the heating mantle at 50 °C. This indicated that no nanoparticle formation occurred and that the polymer chains were fully soluble. However, upon cooling to room temperature, all solutions except PNAM_40_*-b-*PCAm_50_ became turbid, with cloudiness increasing with PCAm DP. This signified that the PCAm block had become insoluble, and spontaneous self-assembly into nanoparticles had occurred. DLS measurements (Figure S2) confirmed the presence of nanoparticles with sizes increasing with increasing DP (*D*_h_ = 44, 67, and 168 nm for DP 100, 150, and 200, respectively). This unexpected thermoresponsive behavior is attributed to the formation of hydrogen bonds between PCAm chains on cooling to 20 °C (Figure 2A). During the initial polymerization, the elevated temperature required for RAFT-PISA means that a hydrogen bond network between cytosines does not form and the chains remain dissolved.^34^ Upon cooling, these bonds reform, causing an increase in the hydrophobicity of PCAm chains, triggering self-assembly. This indicated that a RAFT solution polymerization took place as opposed to the RAFT dispersion polymerization that occurred at pH 7. Consequently, the phase separation of colloidally unstable nanoparticles during PCAm chain growth seen at pH 7 was not possible. This allowed PCAm chains to fully grow before undergoing postpolymerization self-assembly into nanoparticles as opposed to at pH 7 where *in situ* self-assembly took place. We investigated the reproducibility of the thermoresponsive behavior for PNAM_40_*-b-*PCAm_200_ using DLS (Figure 2B). Nanoparticles consistently disassembled and reformed after 10 heat–cool cycles (*D*_h_ = 145 ± 10 nm at 20 °C), which suggested that they were not kinetically trapped at 20 °C. We then attempted to confirm nanoparticle morphology using TEM; however, upon dilution to an appropriate concentration (0.5 mg ml^–1^) for imaging, the solution became clear, indicating nanoparticle disassembly. This was highly surprising as polymer nanoparticles are typically stable to dilution due to their low critical micelle concentrations and high number of entanglements between polymer chains when self-assembled; this result suggests high chain mobility at pH 2 but not pH 7.^35^

Given that the pH dependence of cytosine association had indeed translated to the self-assembly of polymers, we investigated whether these new self-assembled structures could be trapped in their morphologies. To increase the solution pH to 7, above the p*K*_a_ for cytosine, while minimizing sample dilution, a drop of 1 M sodium hydroxide was added to deprotonate the cytosine units. For PNAM_40_*-b-*PCAm_100_, the solution became significantly more turbid after increasing the pH indicating an increase in nanoparticle size, which was further confirmed by DLS (Figure S3). Macroscopic precipitation was observed upon the addition of hydroxide to PNAM_40_*-b-*PCAm_150_, preventing further analysis. Finally, PNAM_40_*-b-*PCAm_200_ showed no visible change upon basification, and DLS analyses showed no change in the particle size (Figure 2C). The neutral PNAM_40_*-b-*PCAm_200_ particles were now stable to dilution, and TEM images showed a pure vesicular morphology (Figure 2D), a phase that was previously not accessible at neutral pH before. As there was no change to the particle size upon basification, we assumed that the polymers also assembled into vesicles under acidic conditions. We surmised that upon basification, the amphiphilic polymer chains are not trapped in their existing self-assembled states but will preferentially reorganize to a thermodynamically favored morphology. For PNAM_40_*-b-*PCAm_150_, this rearrangement of polymer chains is likely hindered by the poor mobility of the polymer chains in solution leading to precipitation similar to experiments performed when building our initial PCAm phase diagram at neutral pH. No precipitation is seen for PNAM_40_*-b-*PCAm_200_ because the chains are already in their preferred morphology; therefore, no significant chain reorganization occurs. While reacidification of a diluted vesicle solution led to nanoparticle dissolution, similar to our observations when diluting vesicles before any pH change, we expect that as long as no significant dilution occurs when altering pH, the dynamic behavior of these vesicles can be reversibly switched.

**Figure 3:**
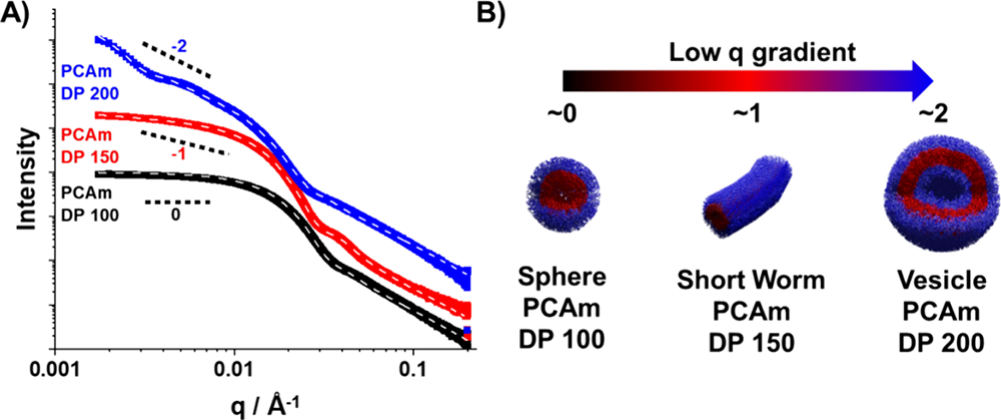
(A) Background-subtracted SAXS patterns and data fits (dotted line) obtained for 2.5% w/w PNAM_40_*-b-*PCAm_*x*_ copolymer dispersions in pH 2 water at 20 °C. Low *q* gradients of 0, −1 and −2 are shown as a guide to the eye and represent the expected slopes for spheres, worms, and vesicles, respectively. (B) Schematic illustration of the correlation between the low *q* gradient in SAXS data and polymer nanoparticle morphology.

To further confirm the morphology and study the self-assembly of these polymers at pH 2, which was not possible with TEM due to the disassembly of particles upon dilution, synchrotron small-angle X-ray scattering (SAXS) studies were performed.^36^ Initially, static measurements at 20 °C were performed to confirm the nanoparticle morphology (Figure 3A). The background-subtracted radially integrated patterns obtained for these three PNAM_40_*-b-*PCAm_*x*_ dispersions are plotted as the X-ray scattering intensity, *I*(*q*), versus the scattering vector, *q*. Predominant copolymer morphology can be initially estimated by the gradient in the low *q* region of the SAXS pattern (Figure 3B).^37^ Spherical micelles exhibit a low *q* gradient of 0, worm-like micelles have a gradient of −1, and a gradient of approximately −2 indicates the presence of vesicles. The SAXS pattern for PNAM_40_*-b-*PCAm_100_ (Table S2) was satisfactorily fitted using a spherical micelle model (Figure S4A),^38^ which indicated a sphere volume-average diameter of 30 nm. For PNAM_40_*-b-*PCAm_150_ (Table S3)_,_ while the low *q* gradient appears closer to zero than −1, indicating a spherical morphology, we found that these SAXS patterns could be best fitted using a worm-like micelle model (Figure S4B).^38^ Attempts to model the data using a pure spherical micelle model^38^ and a sphere, dimer, and trimer model^39^ were unsuccessful in obtaining satisfactory fits in later temperature sweep experiments; thus the worm-like micelle model was used throughout for consistency. As such, we classified these nanoparticles as short worms with a determined cross-sectional volume-average diameter and mean particle length of 30 and 43 nm, respectively. Intriguingly for PNAM_40_*-b-*PCAm_200_ (Table S4), despite TEM images indicating a pure vesicle phase, the SAXS pattern obtained could only be satisfactorily fitted when using a combined worm and vesicle model (Figure S4C).^40^ The model indicated short worms with a cross-sectional diameter and length of 30 and 43 nm, respectively, that were present along with a predominant vesicle phase with a volume-average diameter of 206 nm and a mean vesicle membrane thickness of 26 nm. In all cases, fitting SAXS data indicated a small amount of molecularly dissolved copolymer chains, which were accounted for using a Gaussian chain model (Figure S4).^41^

**Figure 4:**
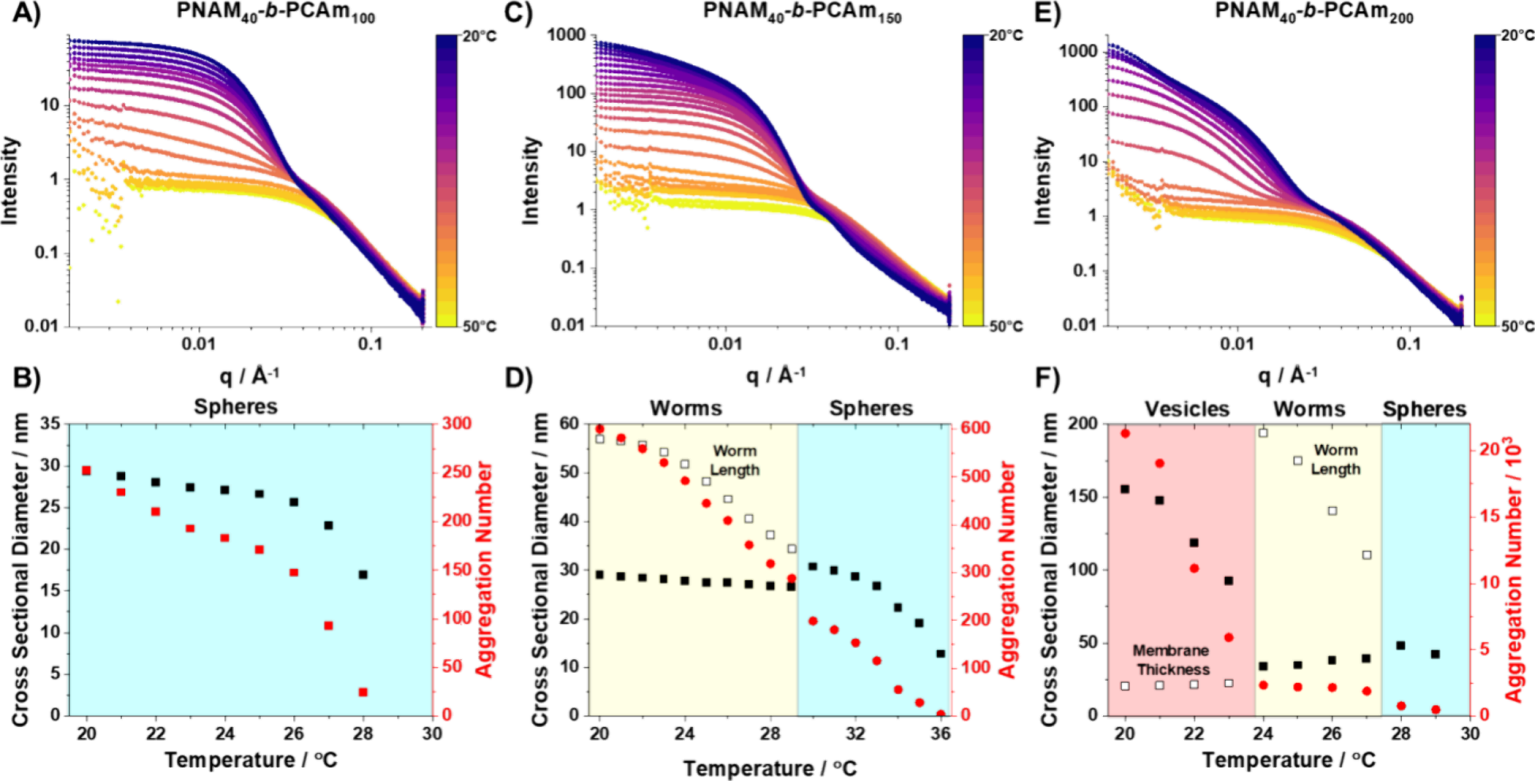
Variable temperature background-subtracted SAXS patterns recorded for 2.5% w/w PNAM_40_-*b*-PCAm_*x*_ copolymer dispersions in pH 2 water, PCAm DP = (A) 100, (C) 150, and (E) 200, during a cooling cycle (50 → 20 °C) at a cooling rate of 1 °C/min. Evolution of nanoparticle cross-sectional diameter (filled square), aggregation number (filled circle), and additional nanoparticle features (open square) with temperature for PCAm DP = (B) 100, (D) 150, and (F) 200. Shaded sections are used to highlight the predominant morphology present: either spheres (blue), worms (yellow), or vesicles (red).

While static SAXS measurements enabled us to confirm the solution state morphologies of our polymers at 20 °C, it did not elucidate how the polymer chains self-assembled upon cooling. We performed variable temperature SAXS measurements in which polymer solutions were heated to 50 °C, at which point only free polymer chains were present. Then samples were steadily cooled down to 20 °C at 1 °C/min while collecting scattering data. For all scattering patterns modeled, free polymer chains were present, indicating that not all chains self-assembled into nanoparticles. For PNAM_40_*-b-*PCAm_100_ (Figure 4A,B), only free chains could be observed on cooling from 50 to 28 °C at which point spherical particles of ∼17 nm in diameter began to form. Upon further cooling, these spherical particles rapidly grew in size to 30 nm, the same as the initial static measurements. Additionally, the number of copolymer chains assembled per nanoparticle, or particle aggregation number (*N*_agg_), steadily increased upon cooling with a minimal increase in particle size, likely indicating rearrangement and equilibration of chains within the particle. For PNAM_40_*-b-*PCAm_150_ (Figure 4C,D), particle nucleation occurred at a higher temperature (36 °C) compared to PNAM_40_*-b-*PCAm_100_. On cooling below 36 °C, the initial particle growth was similar to PNAM_40_*-b-*PCAm_100_ with spherical nanoparticles being formed. However, below 29 °C, scattering patterns were better fitted using a worm-like micelle model. These short worms continued to grow in length up to 57 nm, while their width consistently remained around ∼29 nm, comparable to the final mean sphere diameter. These observations are fully consistent with a worm growth mechanism based on the stochastic 1D fusion of multiple spheres.^42,43^ Comparing *N*_agg_ and the size of the final short worms to spheres before fusion, it is likely that these short worms are composed of two spheres and thus are more adequately described as dimers. Finally, for PNAM_40_*-b-*PCAm_200_ (Figure 4E,F), particle nucleation occurred at 29 °C to form spherical nanoparticles that rapidly fused to form worm-like micelles as the temperature further decreased. Interestingly, these worms were much longer (111–196 nm) than those observed for PNAM_40_*-b-*PCAm_150_. As the temperature was further decreased to 23 °C, the scattering patterns were best fit using a mixed worm and vesicle model, with vesicles being the predominant phase. Upon further cooling, the worm size remained consistent. However, the size of vesicles increased significantly up to 155 nm and *N*_agg_ also increased to 21,000 at 20 °C. Additionally, once the vesicles had formed, their membrane thickness remained consistently around ∼20 nm, slightly smaller than the size of the hydrophobic core of the formed worms likely indicating interdigitation of PCAm chains.^44^ It is worth noting that these vesicles never reached the size that was achieved in the initial static measurement. Furthermore, the volume fraction of vesicles within the hybrid SAXS model fit at the end of the temperature sweep (29%) was also smaller than the initial static measurements (51%). Given these observations, it is likely the time scale of this experiment was not sufficient to observe the final stage of the reformation process. Typically, during PISA, an increasing chain length causes phase transitions. Instead of changing the chain length, here, we use stimuli to modulate the hydrophobicity of the fully formed chain. While this system has a fixed chain length of the core-forming block, the increasing PCAm hydrophobicity due to hydrogen bond reformation upon cooling is clearly analogous to the PISA process.

## Conclusions

In summary, we successfully chain-extended a PNAM_40_ mCTA via the aqueous RAFT-PISA of cytosine acrylamide. A phase diagram was constructed with pure spherical and worm phases as well as mixed phases observed. Furthermore, we investigated the stimuli-responsive self-assembly behavior of cytosine acrylamide under conditions, which could allow i-motif formation. The polymers displayed orthogonal stimuli responsiveness (temperature and pH), which affected the nanoparticle stability. During the polymerizations at 50 °C, no nanoparticles were produced owing to the increased hydrophilicity of the protonated PCAm_40_ chains and disruption of hydrogen bonding at elevated temperatures. Upon cooling, hydrogen bonds reformed, inducing amphiphilicity in the polymer and thus driving self-assembly, even for PCAm DPs that were previously inaccessible when polymerizing at pH 7. For these nanoparticles, high unimer mobility was evident, a trait usually associated with assemblies formed of small molecules rather than polymers. However, these particles could be made stable to dilution by increasing solution pH after self-assembly. Static SAXS measurements confirmed the formation of spheres, short worms, and mixed worm-vesicle phases for PCAm DPs of 100, 150, and 200, respectively. Variable temperature SAXS experiments indicated that PNAM_40_*-b-*PCAm_150_ and PNAM_40_*-b-*PCAm_200_ assemblies accessed higher-order assemblies through the same morphological pathway as that of PISA systems.

## Supporting Information Available

GPC and DLS characterization for PNAM-*b*-PCAm polymerizations at pH 7, DLS characterization for PNAM-PCAm polymerizations at pH 2, and temperature-responsive small-angle X-ray scattering analysis of PNAM-*b*-PCAm polymers (PDF)
