# Supplementary material for: Harnessing Cytosine for Tunable Nanoparticle Self-Assembly Behavior Using Orthogonal Stimuli
Source: Biomacromolecules. 2024 Jul 15;25(8):4905–12. doi: 10.1021/acs.biomac.4c00352 (PMC11323014; doi:10.1021/acs.biomac.4c00352)
Supplement: Supplementary file 1 — bm4c00352_si_001.pdf [file bm4c00352_si_001.pdf]

Supplementary information for Harnessing Cytosine for Tunable Nanoparticle Self-Assembly Behaviour Using Orthogonal Stimuli

Sam J. Parkinson[a], Stephen D. P. Fielden[a], Marjolaine Thomas[b], Alisha J. Miller[a], Paul D. Topham[b], Matthew J. Derry[b], Rachel K. O'Reilly[a]*

SAXS data modelling

Programming tools within the Irena SAS Igor Pro macros[1] were used to implement the scattering models.

In general, the intensity of X-rays scattered by a dispersion of nano-objects [as represented by the scattering cross-section per unit sample volume, $\frac{d\Sigma}{d\Omega}(q)$] can be expressed as:

$$\frac{d\Sigma}{d\Omega}(q) = NS(q)\int_0^\infty \dots \int_0^\infty F(q, r_{1,} \dots, r_k)^2 \Psi(r_{1,} \dots, r_k) dr_{1,} \dots, dr_k \qquad \text{(S1)}$$

where $F(q, r_1, \dots, r_k)$ is the form factor, $r_{1,} \dots, r_k$ is a set of *k* parameters describing the structural morphology, $\Psi(r_{1,} \dots, r_k)$ is the distribution function, *S*(*q*) is the structure factor and *N* is the number density of nano-objects per unit volume expressed as:

$$N = \frac{\varphi}{\int_0^\infty \dots \int_0^\infty V(r_1, \dots, r_k)\Psi(r_1, \dots, r_k) dr_1, \dots, dr_k} \qquad \text{(S2)}$$

where $V(r_1, \dots, r_k)$ is the volume of the nano-object and φ is its volume fraction within the dispersion. It is assumed that *S*(*q*) = 1 in this study.

Spherical micelle model

The spherical micelle form factor for Equation S1 is given by[2]:

$$F_{s_{mic}}(q) = N_s{}^2\beta_s{}^2 A_s{}^2(q, R_s) + N_s\beta_c{}^2 F_c(q, R_g) + N_s(N_s - 1)\beta_c{}^2 A_c{}^2(q) + 2N_s{}^2\beta_s\beta_c A_s(q, R_s)A_c(q) \qquad \text{(S3)}$$

where R_s is the volume-average sphere core radius and R_g is the radius of gyration of the coronal steric stabilizer block (in this case, $PHEMA_{30}$). The X-ray scattering length contrasts for the core and corona blocks are given by $\beta_s = V_s(\xi_s - \xi_{sol})$ and $\beta_c = V_c(\xi_c - \xi_{sol})$ respectively. Here, ξ_s, ξ_c and ξ_{sol} are the X-ray scattering length densities of the core block (ξ_{PCAm}= 10.63 x 10^{10} cm^{-2}), corona block (ξ_{PNAM}= 10.60 x 10^{10} cm^{-2}) and pH2 water solvent (ξ_{sol}= 9.42 x 10^{10} cm^{-2}), respectively. V_s and V_c are the volumes of the core block (V_{PCAm}) and the corona block (V_{PNAM}), respectively. The sphere form factor amplitude is used for the amplitude of the core self-term:

$$A_c(q, R_s) = \Phi(qR_s)\exp\left(-\frac{q^2\sigma^2}{2}\right) \qquad \text{(S4)}$$

where $\Phi(qR_s) = \frac{3[\sin(qR_s) - qR_s\cos(qR_s)]}{(qR_s)^3}$. A sigmoidal interface between the two blocks was assumed for the spherical micelle form factor (Equation S3). This is described by the exponent term with a width σ accounting for a decaying scattering length density at the micellar interface. This σ value was fixed at 2.5 during fitting.

The form factor amplitude of the spherical micelle corona is:

$$A_c(q) = \frac{\int_{R_s}^{R_s+2s} \mu_c(r) \frac{\sin(qr)}{qr} r^2 dr}{\int_{R_s}^{R_s+2s} \mu_c(r) r^2 dr} exp\left(-\frac{q^2\sigma^2}{2}\right) \qquad (S5)$$

The radial profile, $\mu_c(r)$, can be expressed by a linear combination of two cubic bsplines, with two fitting parameters s and a corresponding to the width of the profile and the weight coefficient respectively. This information can be found elsewhere,[3, 4] as can the approximate integrated form of Equation S5. The self-correlation term for the coronal block is given by the Debye function:

$$F_c(q, R_g) = \frac{2\left[\exp\left(-q^2{R_g}^2\right) - 1 + q^2{R_g}^2\right]}{q^4{R_g}^4} \qquad (S6)$$

where R_g is the radius of gyration of the PNAM coronal block. In all cases R_g was fixed to be 1.6 nm, which is estimated by assuming the total contour length of $PNAM_{40}$ is 10.21 nm (40 × 0.255 nm, where 0.225 nm is the contour length of one NAM monomer unit with two C-C bonds in all-trans conformation). Given a mean Kuhn length of 1.53 nm, based on the known literature value for poly(methyl methacrylate)[5], an estimated unperturbed R_g of 1.6 nm is determined using R_g = $(10.21 \times 1.53/6)^{0.5}$.

The aggregation number, N_s, of the spherical micelle is given by:

$$N_s = (1 - x_{sol}) \frac{\frac{4}{3}\pi {R_s}^3}{V_s} \qquad (S7)$$

where x_{sol} is the volume fraction of solvent within the PCAm micelle cores, which was found to be zero in all cases. A polydispersity for one parameter (R_s) is assumed for the micelle model, which is described by a Gaussian distribution. Thus, the polydispersity function in Equation S1 can be represented as:

$$\Psi(r_1) = \frac{1}{\sqrt{2\pi{\sigma_{Rs}}^2}} exp\left(-\frac{(r_1 - R_s)^2}{2{\sigma_{Rs}}^2}\right) \qquad (S8)$$

where σ_{Rs} is the standard deviation for R_s. In accordance with Equation S2, the number density per unit volume for the micelle model is expressed as:

$$N = \frac{\varphi}{\int_0^\infty V(r_1)\Psi(r_1)dr_1} \qquad (S9)$$

where φ is the total volume fraction of copolymer in the spherical micelles and $V(r_1)$ is the total volume of copolymer within a spherical micelle $[V(r_1) = (V_s + V_c)N_s(r_1)]$.

Worm-like micelle model

The worm-like micelle form factor for Equation S1 is given by:

$$F_{w\_mic}(q) = N_w{}^2\beta_s{}^2F_{sw}(q) + N_w\beta_c{}^2F_c(q, R_g) + N_w(N_w - 1)\beta_c{}^2S_{cc}(q) + 2N_w{}^2\beta_s\beta_cS_{sc}(q) \quad \text{(S10)}$$

where all the parameters are the same as those described in the spherical micelle model (Equation S3), unless stated otherwise.

The self-correlation term for the worm core cross-sectional volume-average radius R_w is:

$$F_{sw}(q) = F_{worm}(q, L_w, b_w)A_{CS_{worm}}{}^2(q, R_w) \quad \text{(S11)}$$

where

$$A_{CS_{worm}}{}^2(q, R_w) = \left[2\frac{J_1(qR_w)}{qR_w}\right]^2 \quad \text{(S12)}$$

and J_1 is the first-order Bessel function of the first kind, and a form factor $F_{worm}(q, L_w, b_w)$ for self-avoiding semi-flexible chains represents the worm-like micelles, where b_w is the Kuhn length and L_w is the mean contour length. A complete expression for the chain form factor can be found elsewhere. [6]

The mean aggregation number of the worm-like micelle, N_w, is given by:

$$N_w = (1 - x_{sol})\frac{\pi R_w{}^2L_w}{V_s} \quad \text{(S13)}$$

where x_{sol} is the volume fraction of solvent within the worm-like micelle cores, which was found to be zero in all cases. The possible presence of semi-spherical caps at both ends of each worm is neglected in this form factor.

A polydispersity for one parameter (R_w) is assumed for the micelle model, which is described by a Gaussian distribution. Thus, the polydispersity function in Equation S1 can be represented as:

$$\Psi(r_1) = \frac{1}{\sqrt{2\pi\sigma_{R_w}{}^2}}exp\left(-\frac{(r_1 - R_w)^2}{2\sigma_{R_w}{}^2}\right) \quad \text{(S14)}$$

where σ_{R_w} is the standard deviation for R_w. In accordance with Equation S2, the number density per unit volume for the worm-like micelle model is expressed as:

$$N = \frac{\varphi}{\int_0^\infty V(r_1)\Psi(r_1)dr_1} \quad \text{(S15)}$$

where φ is the total volume fraction of copolymer in the worm-like micelles and $V(r_1)$ is the total volume of copolymer in a worm-like micelle $[V(r_1) = (V_s + V_c)N_w(r_1)]$.

Vesicle model

The vesicle form factor in Equation S1 is expressed as:[7]

$$F_{ves}(q) = N_v{}^2\beta_m{}^2A_m{}^2(q) + N_v\beta_{vc}{}^2F_c(q,R_g) + N_v(N_v - 1)\beta_{vc}{}^2A_{vc}{}^2(q) + 2N_v{}^2\beta_m\beta_{vc}A_m(q)A_{vc}(q) \quad \text{(S16)}$$

where all the parameters are the same as in the spherical micelle model (see Equation S3) unless stated otherwise.

The amplitude of the membrane self-term is:

$$A_m(q) = \frac{V_{out}\varphi(qR_{out}) - V_{in}\varphi(qR_{in})}{V_{out} - V_{in}} exp\left(-\frac{q^2\sigma_{in}{}^2}{2}\right) \quad \text{(S17)}$$

where $R_{in} = R_m - \frac{1}{2}T_m$ is the inner radius of the membrane, $R_{out} = R_m + \frac{1}{2}T_m$ is the outer radius of the membrane (R_m is the radius from the centre of the vesicle to the centre of the membrane), $V_{in} = \frac{4}{3}\pi R_{in}{}^3$ and $V_{out} = \frac{4}{3}\pi R_{out}{}^3$. It should be noted that Equation S16 differs subtly from the original work in which it was first described.[7] The exponent term in Equation S17 represents a sigmoidal interface between the blocks, with a width σ_{in} accounting for a decaying scattering length density at the membrane surface. The value of σ_{in} was fixed at 2.5 during fitting. The mean vesicle aggregation number, N_v, is given by:

$$N_v = (1 - x_{sol})\frac{V_{out} - V_{in}}{V_m} \quad \text{(S18)}$$

where x_{sol} is the volume fraction of solvent within the vesicle membrane, which was found to be zero in all cases. Assuming that there is no penetration of the solvophilic coronal blocks into the solvophobic membrane, the amplitude of the vesicle corona self-term is expressed as:

$$A_{vc}(q) = \Psi(qR_g)\frac{1}{2}\left[\frac{sin[q(R_{out} + R_g)]}{q(R_{out} + R_g)} + \frac{\sin[q(R_{in} - R_g)]}{q(R_{in} - R_g)}\right] \quad \text{(S19)}$$

where the term outside the square brackets is the factor amplitude of the corona block polymer chain such that:

$$\Psi(qR_g) = \frac{1 - \exp(-qR_g)}{(qR_g)^2} \quad \text{(S20)}$$

For the vesicle model, it was assumed that two parameters are polydisperse: the radius from the centre of the vesicles to the centre of the membrane and the membrane thickness (denoted R_m and

T_m, respectively). Each parameter is considered to have a Gaussian distribution of values, so the polydispersity function in Equation S1 can be expressed in each case as:

$$\Psi(r_1 r_2) = \frac{1}{\sqrt{2\pi\sigma_{Rs}{}^2}} exp\left(-\frac{(r_1 - R_m)^2}{2\sigma_{Rm}{}^2}\right) \frac{1}{\sqrt{2\pi\sigma_{Tm}{}^2}} exp\left(-\frac{(r_1 - T_m)^2}{2\sigma_{Tm}{}^2}\right) \quad \text{(S21)}$$

where σ_{Rm} and σ_{Tm} are the standard deviations for R_m and T_m, respectively. Following Equation S2, the number density per unit volume for the vesicle model is expressed as:

$$N = \frac{\varphi}{\int_0^\infty \int_0^\infty V(r_1, r_2)\, \Psi(r_1, r_2) dr_1 dr_2} \quad \text{(S22)}$$

where φ is the total volume fraction of copolymer in the vesicles and $V(r_1, r_2)$ is the total volume of copolymers in a vesicle $[V(r_1, r_2) = (V_m + V_{vc})N_v(r_1, r_2)]$.

Gaussian chain model

Generally, the scattering cross-section per unit sample volume for an individual Gaussian polymer chain can be expressed as:[8]

$$\frac{d\Sigma}{d\Omega}(q) = \varphi(\Delta\xi)^2 V_{\mathrm{mol}} F_{\mathrm{mol}}(q) \quad \text{(S23)}$$

where V_{mol} is the total molecular volume and $\Delta\xi$ is the excess scattering length density of the copolymer [$\Delta\xi = \xi_{\mathrm{PNAM-PCAm}} - \xi_{\mathrm{water}} = 1.18 \times 10^{-10}$ cm^{-2}], where the scattering length density of the copolymer is calculated as $\xi_{\mathrm{PNAM-PCAm}} = \frac{V_{\mathrm{PNAM}}\xi_{\mathrm{PNAM}} + V_{\mathrm{PCAm}}\xi_{\mathrm{PCAm}}}{V_{\mathrm{PNAM-PCAm}}}$. The generalised form factor for a Gaussian polymer chain is given by:

$$F_{\mathrm{mol}}(q) = \left[\frac{1}{\upsilon U^{1/(2\upsilon)}} \gamma\left(\frac{1}{2\upsilon}, U\right) - \frac{1}{\upsilon U^{1/\upsilon}} \left(\frac{1}{\upsilon}, U\right)\right] \quad \text{(S24)}$$

where the lower incomplete gamma function is $\gamma(s, x) = \int_0^x t^{s-1} \exp(-t)\, dt$ and U is the modified variable:

$$U = (2\upsilon + 1)(2\upsilon + 2)\frac{q^2 R_{g_{\mathrm{cop}}}{}^2}{6} \quad \text{(S25)}$$

Here, υ is the extended volume parameter and $R_{g_{\mathrm{cop}}}$ is the radius of gyration of the copolymer chain.

Table S1. Characterisation data obtained for $PNAM_{40}$-*b*-$PCAm_x$ nanoparticles

Solids Content (% w/w)	Targeted DP	% Conv.	$M_{n,SEC}$ (kDa)	$Đ_{M,SEC}$	D_h (nm)	PD	Morphology
2.5	10	> 99	10.6	1.27	-[a]	-[a]	S
	50	> 99	18.5	1.18	42	0.30	S+W
	100	> 99	30.5	1.08	294	0.10	S+W
5	10	> 99	10.7	1.12	-[a]	-[a]	S
	50	> 99	17.1	1.38	32	0.27	S+W
	100	> 99	20.2	1.47	-[a]	-[a]	W
10	10	> 99	10.3	1.18	117	0.43	S
	50	> 99	14.4	1.20	180	0.03	S+W
	100	> 99	26.4	1.35	-[a]	-[a]	S+W+V

[a] No suitable measurement data could be obtained via DLS

S – spheres , S+W – spheres and worms, W – worms, S+W+V – spheres, worms & vesicles

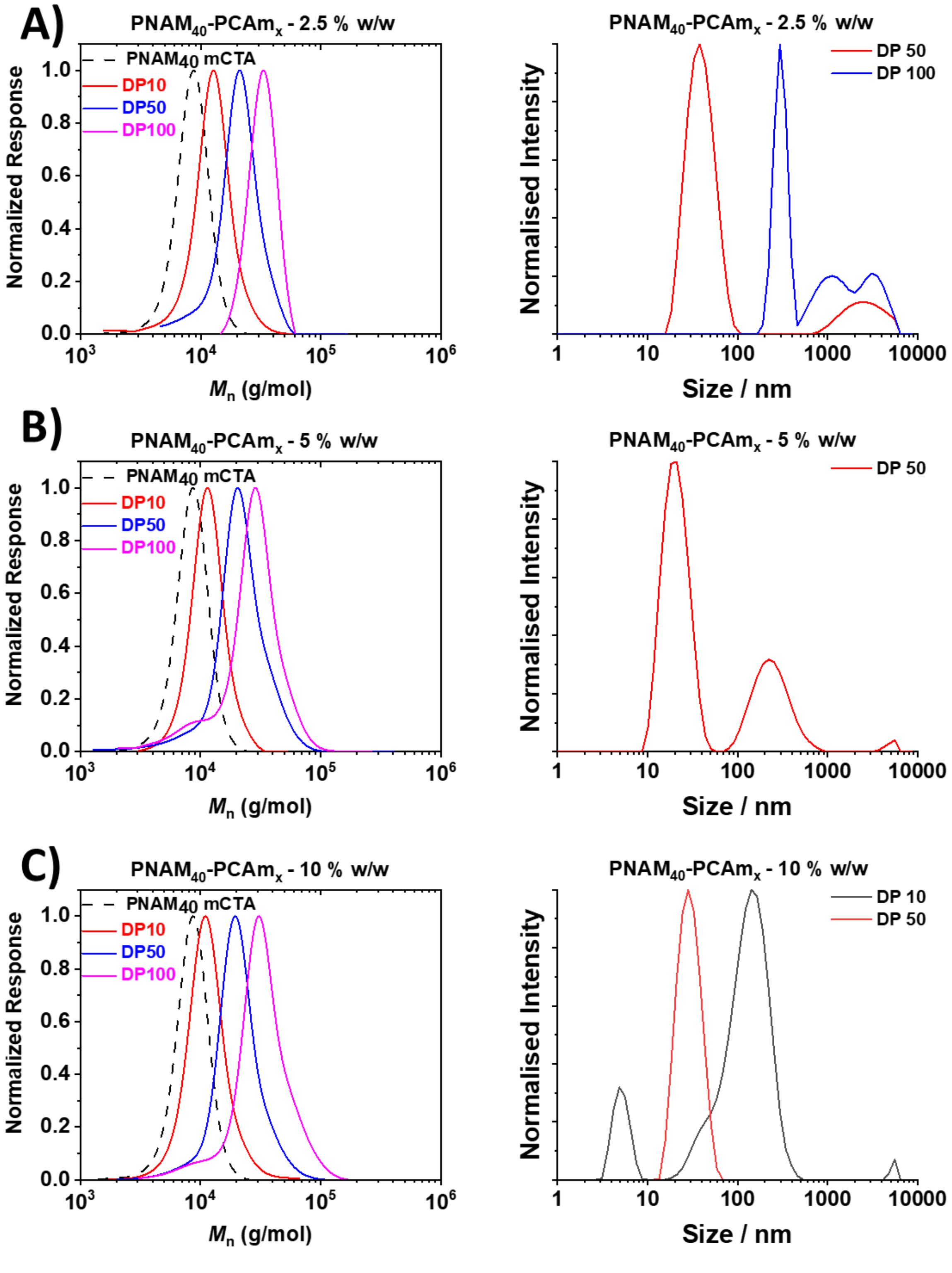

Figure S1. GPC and DLS characterisation data obtained for $PNAM_{40}$-*b*-$PCAm_x$ nanoparticles at a) 2.5 % w/w, b) 5% w/w and c) 10 % w/w respectively. All polymerisations were carried out at pH 7.

Table S2. Modelled SAXS scattering pattern data obtained for $PNAM_{40}$-*b*-$PCAm_{100}$ nanoparticles. S = spherical micelles.

Temperature (°C)	Cross-Sectional Diameter (nm)	Aggregation number (N_{agg})	Volume Fraction of S (%)	Fitting Model
28	17	24	100	S
27	23	92	100	S
26	26	147	100	S
25	27	171	100	S
24	27	183	100	S
23	27	192	100	S
22	28	210	100	S
21	29	230	100	S
20	29	252	100	S
Static	30	268	100	S

Table S3. Modelled SAXS scattering pattern data obtained for PNAM$_{40}$-*b*-PCAm$_{150}$ nanoparticles. S = spherical micelles; W = worm-like micelles.

Temperature (°C)	Cross-Sectional Diameter (nm)	Worm Length (nm)	Aggregation number (N_{agg})	Volume Fraction (%)		Fitting Model
				S	W	
36	13	-	3	100	0	S
35	19	-	28	100	0	S
34	22	-	55	100	0	S
33	27	-	115	100	0	S
32	29	-	153	100	0	S
31	30	-	180	100	0	S
30	31	-	198	100	0	S
29	26	34	318	100	0	S
28	27	37	318	100	0	S
27	27	41	358	0	100	W
26	27	45	409	0	100	W
25	27	48	445	0	100	W
24	28	52	492	0	100	W
23	28	54	530	0	100	W
22	28	56	558	0	100	W
21	29	57	581	0	100	W
20	29	57	600	0	100	W
Static	30	43	493	0	100	W

Table S4. Modelled SAXS scattering pattern data obtained for $PNAM_{40}$-*b*-$PCAm_{200}$ nanoparticles. S = spherical micelle; W = worm-like micelle; V = vesicle.

Temperature (°C)	Cross-Sectional Diameter (nm)	Worm Length (nm)	Membrane Thickness (nm)	Aggregation number (N_{agg})	Volume Fraction (%)			Fitting Model
					S	W	V	
29	42	-	-	480	100	0	0	S
28	48	-	-	760	79	21	0	S + W
27	39	111	-	1873	0	100	0	W
26	38	141	-	2155	0	100	0	W
25	35	175	-	2185	0	100	0	W
24	34	194	-	2319	0	66	34	W + V
	121	-	21	11685				
23	35	174	-	2268	0	57	43	W + V
	93	-	23	5915				
22	34	196	-	2341	0	66	34	W + V
	118	-	21	11129				
21	33	189	-	2118	0	73	27	W + V
	148	-	21	19053				
20	34	185	-	2136	0	71	29	W + V
	155	-	21	21300				
Static	30	43	-	370	0	49	51	W + V
	206	-	26	49543				

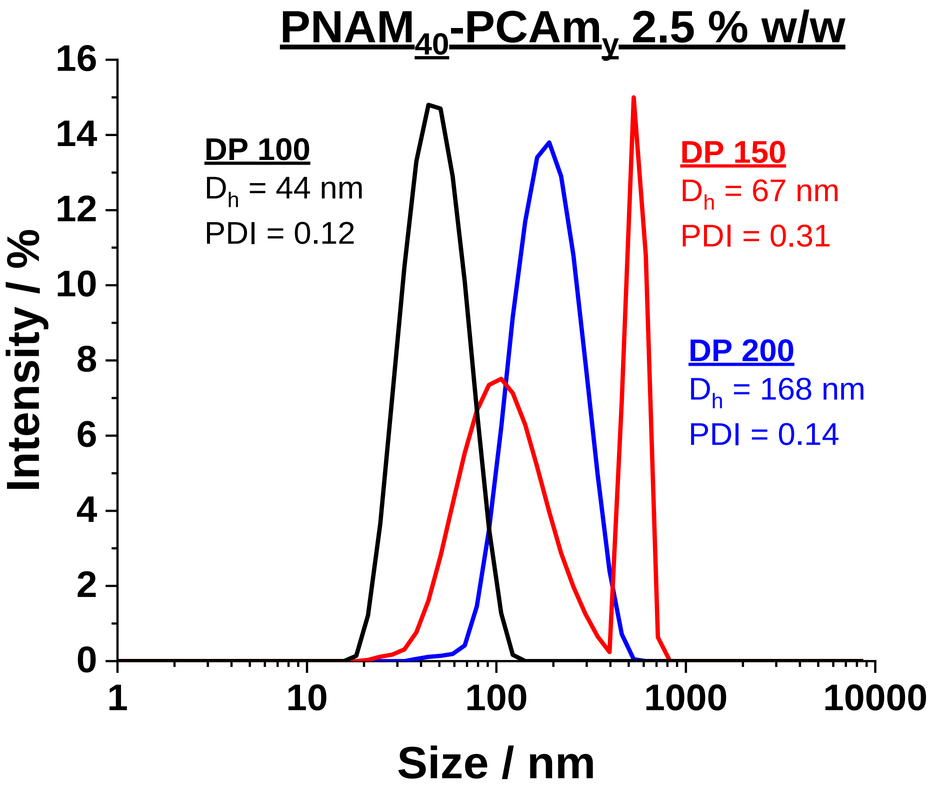

Figure S2. DLS traces for poly(*N*-acryloyl morpholine)$_{40}$-*b*-poly(cytosine acrylamide) nanoparticles formed at pH 2.

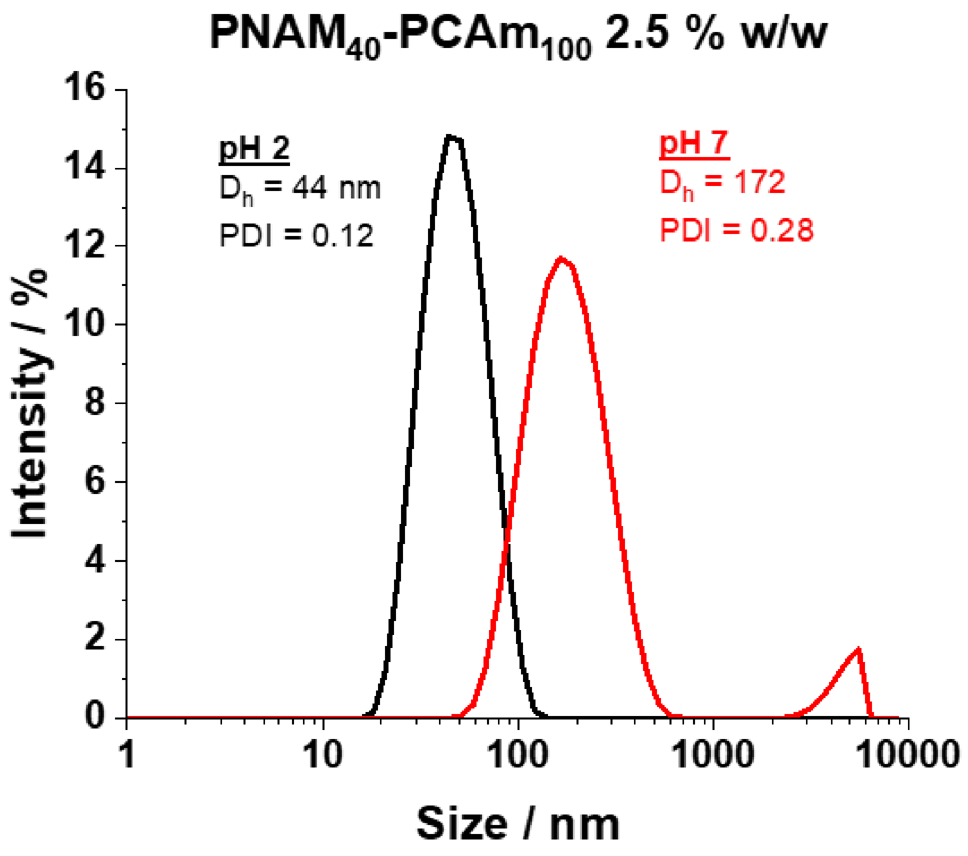

Figure S3. DLS traces for $PNAM_{40}$-*b*-$PCAm_{100}$ nanoparticles before (pH 2) and after basification (pH 7).

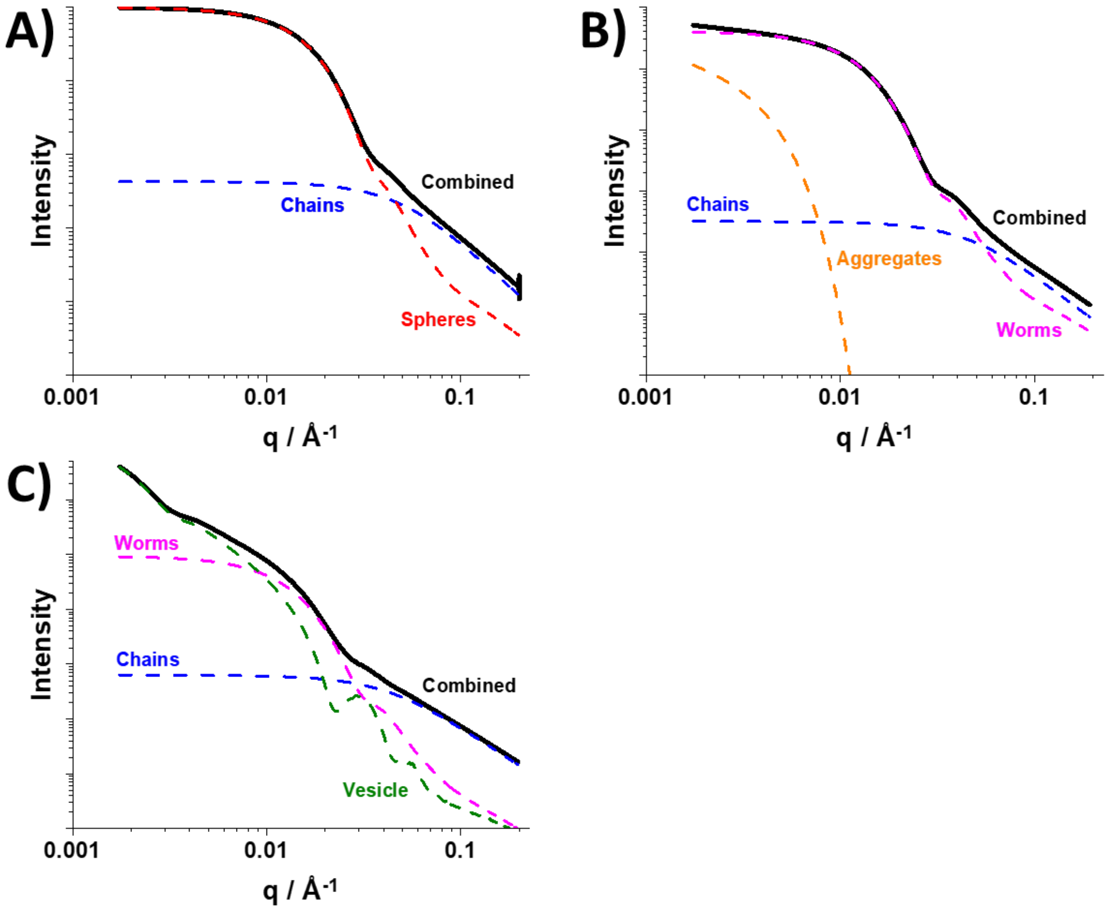

Figure S4. Individual and combined model fits used for a) PNAM$_{40}$-*b*-PCAm$_{100,}$ b) PNAM$_{40}$-*b*-PCAm$_{150}$ and c) PNAM$_{40}$-*b*-PCAm$_{200}$.

References

(1) Ilavsky, J.; Jemian, P. R. Irena: tool suite for modeling and analysis of small-angle scattering. *J. Appl. Crystallogr.* **2009**, *42* (2), 347-353, DOI: 10.1107/S0021889809002222
(2) Pedersen, J. S. Form factors of block copolymer micelles with spherical, ellipsoidal and cylindrical cores. *J. Appl. Crystallogr.* **2000**, *33* (1), 637-640, DOI: Doi 10.1107/S0021889899012248
(3) Pedersen, J. S.; Gerstenberg, M. C. The structure of P85 Pluronic block copolymer micelles determined by small-angle neutron scattering. *Colloids Surf., A* **2003**, *213* (2-3), 175-187, DOI: 10.1016/S0927-7757(02)00511-3
(4) Pedersen, J. S.; Svaneborg, C.; Almdal, K.; Hamley, I. W.; Young, R. N. A small-angle neutron and X-ray contrast variation scattering study of the structure of block copolymer micelles: Corona shape and excluded volume interactions. *Macromolecules* **2003**, *36* (2), 416-433, DOI: 10.1021/ma0204913
(5) Fetters, L. J.; Lohse, D. J.; Colby, R. H. *Chain Dimensions and Entanglement Spacings: Datasheet from · Volume : "Physical Properties of Polymers Handbook" in SpringerMaterials (https://doi.org/10.1007/978-0-387-69002-5_25)*; Springer Science+Business Media, LLC.
(6) Pedersen, J. S.; Schurtenberger, P. Scattering functions of semiflexible polymers with and without excluded volume effects. *Macromolecules* **1996**, *29* (23), 7602-7612, DOI: DOI 10.1021/ma9607630
(7) Bang, J.; Jain, S.; Li, Z.; Lodge, T. P.; Pedersen, J. S.; Kesselman, E.; Talmon, Y. Sphere, Cylinder, and Vesicle Nanoaggregates in Poly(styrene-b-isoprene) Diblock Copolymer Solutions. *Macromolecules* **2006**, *39* (3), 1199-1208, DOI: 10.1021/ma052023+
(8) Hammouda, B. Probing Nanoscale Structures – the sans Toolbox. 2008.
